# Supplementary figures and images for: Non-canonical function of an Hif-1α splice variant contributes to the sustained flight of locusts
Source: eLife. 2022 Aug 30;11:e74554. doi: 10.7554/eLife.74554 (PMC9427102; doi:10.7554/eLife.74554)

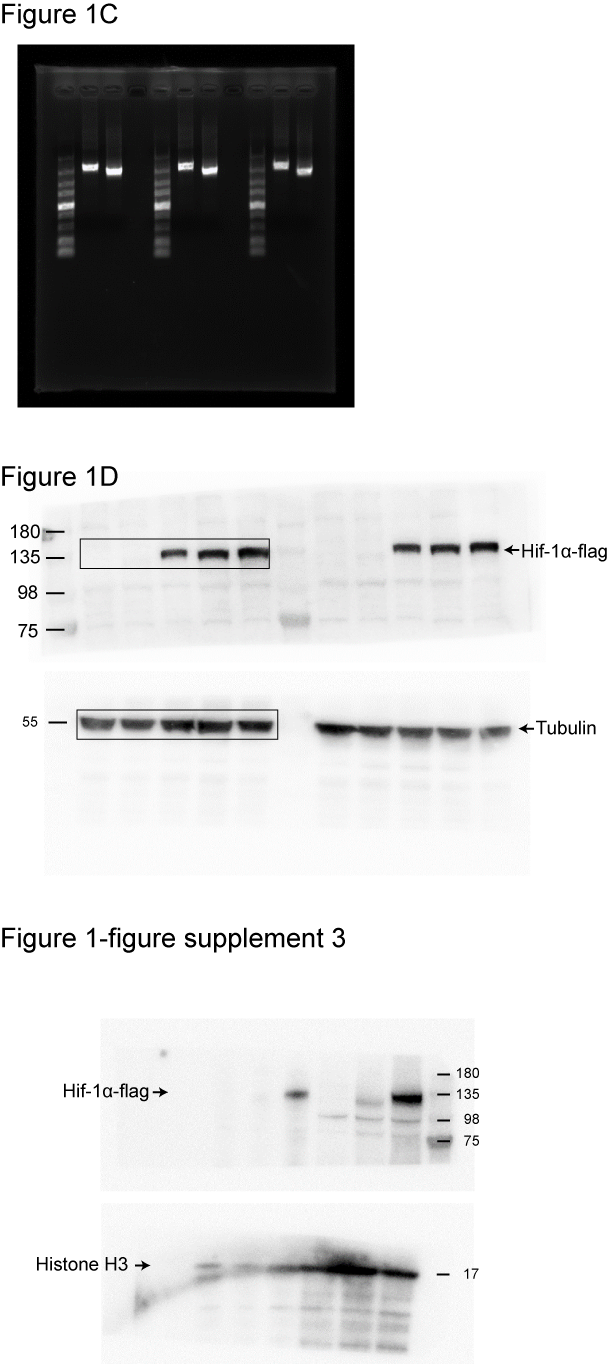

Supplement: Figure 1—source data 2. [file elife-74554-fig1-data2.docx]
