# Supplementary material for: Non-canonical function of an Hif-1α splice variant contributes to the sustained flight of locusts
Source: eLife. 2022 Aug 30;11:e74554. doi: 10.7554/eLife.74554 (PMC9427102; doi:10.7554/eLife.74554)
Supplement: Supplementary file 1. [file elife-74554-supp1.docx]

Primers used in this study

| Primer name | Sequence, 5’-3’ | Description |
| --- | --- | --- |
| Hif-1α-F1 | AAGTATTTAGTGTATATAGC | Clone the full length of *Hif-α1* and *Hif-α2* |
| Hif-1α-F2 | AAGTATTTAGTGTATATAGC |  |
| Hif-1α1-R1 | CATTCAGGAAGTTCAGAATAA |  |
| Hif-1α1-R2 | CTAGTGCTTTAATGGGGTAA |  |
| Hif-1α2-R1 | ATACATTACACCTCTGTTAT |  |
| Hif-1α2-R2 | CTAAGAGGGCTTTGTCTGCT |  |
| Hif-1α1-T7-F | TAATACGACTCACTATAGGAAGAATTCCTTCTCACTTTTG | dsRNA synthesis |
| Hif-1α1-T7-R | TAATACGACTCACTATAGGCATTCAGGAAGTTCAGAATAA |  |
| Hif-1α2-T7-F | TAATACGACTCACTATAGGGTGAGTGCTGGTTACATTTGT |  |
| Hif-1α2-T7-R | TAATACGACTCACTATAGGATACATTACACCTCTGTTAT |  |
| PYK-T7-F | TAATACGACTCACTATAGGATTACAGTAAGAAATTGGGC |  |
| PYK-T7-R | TAATACGACTCACTATAGGGCTGCCGCATTCCGGATGAA |  |
| DJ-1-T7-F | TAATACGACTCACTATAGGTGTTAGCAGAAGGAGCAGAAGA |  |
| DJ-1-T7-R | TAATACGACTCACTATAGGAGGAAGCACCACTACATCGTAT |  |
| PHD-T7-F | TAATACGACTCACTATAGGGAATGGCAGTATTGTCGGAAGT |  |
| PHD-T7-R | TAATACGACTCACTATAGGAAATAGTGGTTCTATATCTG |  |
| Hif-1α-exF | TATCGCGGCCGCGCCATGGCCAGGAATTCGGAGAAACG | Over expression |
| Hif-1α1-exR | TATCTCTAGATTACTTATCGTCGTCATCCTTGTAATCCCCCATTAAAGCACTA |  |
| Hif-1α2-exR | TATCTCTAGATTACTTATCGTCGTCATCCTTGTAATCAGAGGGCTTTGTCTGCT |  |
| DJ-1-gF1 | TATCGAGCTCGAAAAACTCATTTACAGAAT |  |
| DJ-1-gF2 | TATCGAGCTCCAATTTCCGAACATTGATTT |  |
| DJ-1-gR | TATCCTCGAGATACATAATCGCGGAACTTTG |  |
| Hif-1α1-rtF | GGAACGAAAAGCAAGCATAC | qRT-PCR |
| Hif-1α1-rtR | ACTGATAAATTGTGGAATGTGC |  |
| Hif-1α2-rtF | CAGGCTCGACTCGTTCAGATG |  |
| Hif-1α2-rtR | GTGGAAGTGAAACCCAAAGGAA |  |
| DJ-1-rtF | TGCTGCTCCAACTGCTCTGAA |  |
| DJ-1-rtR | CCTGGTCCACGACTTGTGATGA |  |
| LDH-rtF | GTGAAGCACAGCCCAAATAC |  |
| LDH-rtR | GTGACATTAGGAACCGAAAA |  |
| ENO-rtF | ATGCCGCTCAAGAGCTGTCA |  |
| ENO-rtR | ACTCCTTTGCCCATGTAGTT |  |
| PYK-rtF | CAGGCATGAATGTAGCGAGGTT |  |
| PYK-rtR | CCCTTCAAGTAGCCCTGTTCT |  |
| GAPDH-rtF | TGCTGATGCTCCAATGTATGTT |  |
| GAPDH-rtR | ATGGTCCGTCCACAGTCTTC |  |
| GBE1-rtF | GGCAATGGCAATACCTGACA |  |
| GBE1-rtR | CTGATCGTGAGATTCCGCATAT |  |
| MIOX-rtF | TCCGCTTCCACTCGTTCTAC |  |
| MIOX-rtR | CCAGGCAGGTACTTGTCTATGA |  |
| PGI-rtF | ACCGAAGCAATACTCCAATTCT |  |
| PGI-rtR | CAAGTCTGAACCACCAATTCCT |  |
| PGM-rtF | CAAGCAGTCTGGTGTGAAGG |  |
| PGM-rtR | CGGATATGGTCTGAGCCTGT |  |
| PFK-rtF | GCTTGCAGGACAGCGATTA |  |
| PFK-rtR | ACCCATACGACATCCCAAT |  |
| PDHX-rtF | GTCATACGATAACCGAGCCA |  |
| PDHX-rtR | CACTACCGAGCAGCAGTAAAT |  |
| PHD-rtF | CTGAATCGTGACTGGAATGTGA |  |
| PHD-rtR | TCTTGTATGCTGGCTGAACTTC |  |
| PDK-rtF | CTCCCGATTTCTCGTCTGTATG |  |
| PDK-rtR | CGGATGTTTGAACTGTTGCTCC |  |
| RP49-rtF | CGTAAACCGAAGGGAATTGA |  |
| RP49-rtR | GAAGAAACTGCATGGGCAAT |  |
| BNIP3-rtF | GACCGACGACGACCTCAA |  |
| BNIP3-rtR | GCTCCAGTCCCAAATCCA |  |
| GAPDH-gF | TCAGCATTCCTTCCCTTATC | Mitochondrial DNA copy number |
| GAPDH-gR | CAGAGCATTGCGAACTACAT |  |
| CYTB-gF | ATTTCACCTCAGCAACAATA |  |
| CYTB-gR | ACAAGACCAGTTAAACCTCC |  |
| Hif-1α1-F | AAGAATTCCTTCTCACTTTTGGAGC | 3’- RACE |
| Hif-1α2-F | GTGAGTGCTGGTTACATTTGTTTAG |  |
